# Timing of term elective cesarean section and adverse neonatal outcomes: A multi-center retrospective cohort study

**DOI:** 10.1371/journal.pone.0249557

**Published:** 2021-04-05

**Authors:** Ayah Al Bizri, Nansi S. Boghossian, Anwar Nassar, Pascale Nakad, Dina Jaber, Rabih Chahine, Gaby Fallakha, Ghaith Makhoul, Khalid Yunis

**Affiliations:** 1 Department Pediatrics and Adolescent Medicine, National Collaborative Perinatal Neonatal Network, American University of Beirut Medical Center, Beirut, Lebanon; 2 Department of Epidemiology and Biostatistics, Arnold School of Public Health, University of South Carolina, Columbia, South Carolina, United States of America; 3 Department of Obstetrics and Gynecology, American University of Beirut Medical Center, Beirut, Lebanon; 4 Department of Obstetrics and Gynecology, Rafik Hariri University Hospital, Beirut, Lebanon; 5 Department Pediatrics, Centre Hospitalier du Nord, Zgharta, Lebanon; 6 Department of Pediatrics, Notre Dame de la Paix Hospital, Akkar, Lebanon; University of Insubria, ITALY

## Abstract

**Background:**

Rate of cesarean section (CS), including elective CS has globally increased. Studies have found that term elective CS before 39 weeks of gestation is associated with increased risk of adverse respiratory outcomes.

**Objective:**

To determine the rate of elective CS and examine the association between timing of elective term CS and adverse neonatal outcomes in a large population of Lebanese women.

**Methods:**

A Multi-Center Study was conducted using data from the National Collaborative Perinatal Neonatal Network database. Simple and multivariable logistic regression models were used to examine the association between timing of term elective CS and adverse neonatal outcomes. Some of the neonatal adverse outcomes we examined included respiratory distress syndrome, admission to the NICU, and a composite of respiratory outcomes.

**Results:**

A total of 28,997 low risk mothers who delivered through primary and repeat elective CS were included in the study. Uncomplicated elective planned term CS constituted 25% of all CS deliveries in Lebanon. Primary and repeat CS at 37 weeks of gestation increased the odds of most of the studied adverse neonatal outcomes. There were few associations between CS and adverse neonatal outcomes at 38 weeks of gestation.

**Conclusions:**

Term primary and repeat cesarean delivery prior to 39 weeks of gestation is associated with respiratory and other adverse neonatal outcomes. Delaying birth 1–2 weeks till 39 weeks of gestation can prevent 64–77% of adverse respiratory outcomes.

## Introduction

There has been a rise in cesarean section (CS) rates globally [1–3]. The number of newborns delivered through CS has almost doubled increasing from 12% in 2000 to 21% in 2015 [1–4]. Despite the World Health Organization’s recommendation that CS rates should not exceed 10–15% of births, most countries (63%) reported a CS rate above the recommended level [4]. This increase is explained by the rise in both primary and repeat CS [5].

Global elective CS rates vary considerably between different regions and countries. Routine data from the Euro-Peristat study show that the rate of elective CS ranges between 0.5% (Romania) and 38.8% (Cyprus) of total births [6]. Other countries with high elective CS rates include Italy (24.9%), Luxembourg (17.9%), and Malta (16.4%) [6]. In Asia, specifically China, a retrospective cohort study of 66,266 pregnant women showed that between 2007 and 2013, 24.7% underwent elective CS [7]. In the Middle East, varying rates of elective CS have been reported. In Saudi Arabia, 6.3% of 22,595 deliveries between 2008 and 2011 were elective CS (33% of all CS) [8]. In Jordan, an even higher elective CS rate (15.9%) among 21,928 women was reported, representing 29% of all CS deliveries, with the most frequent indication being a previous CS (55.7%) [9].

These high elective CS rates have become a global constant despite an established increased risk of neonatal adverse respiratory morbidities among uncomplicated term pregnancies after elective CS compared to vaginal delivery [10–12]. This risk, however, decreases after 39 weeks of gestation [11]. Accordingly, recent studies have focused on the effect of timing of elective term CS on adverse neonatal outcomes. A secondary analysis of the World Health Organization’s Multicountry Survey on Maternal and Newborn Health, found that among singleton repeat term deliveries, delivery at 37 weeks compared to delivery after 37 weeks increased the odds of neonatal morbidity by two folds (95% CI 1.67–2.56) and intra-hospital early neonatal death by three folds (95% CI 1.72–-6.25) [13]. Moreover, data from the United States, China, and Australia have shown that term elective CS before 39 weeks of gestation significantly increased the risk of adverse respiratory outcomes, admission to the Neonatal Intensive Care Unit (NICU), sepsis, and hospital stay [12,14–17]. Accordingly, postponing delivery to 39 weeks of gestation can potentially decrease the risk of several newborn adverse outcomes.

Studies assessing the effect of timing of elective CS on newborn outcomes are lacking in the Middle East despite the alarmingly high rates of elective CS. Lebanon, a country in the Middle East, has witnessed a tremendous increase in CS from 18% in 2000 to 47% in 2017, a 161% increase over a 18-year time period [18,19]. Although CS rate is very high in Lebanon, the rate of elective CS is unknown. We conducted this study to: (1) determine the rate of elective CS, and (2) examine the association between timing of elective term CS and adverse neonatal outcomes in a large population of Lebanese women.

## Methods

### Data source

The National Collaborative Perinatal Neonatal Network (NCPNN) is a hospital-based surveillance system in Lebanon that collects data on all live births, stillbirths of at least 20 weeks gestation, and outborn neonates transferred to NCPNN member centers within 28 days of life. A total of 33 hospitals submitted data from January 2001 until December 2017; the number of participating hospitals during a given year varied between 10 and 27 hospitals. All data were collected by trained research assistants or local nurses from medical records and direct interviews with the mothers. The NCPNN database undergoes periodic analysis to assess the completeness and quality of the collected data, which is then communicated with the centers to improve the quality of future data collection and when needed retraining of data collectors. Oral consent was obtained from each participating mother. The Institutional Review Board at the American University of Beirut last approved the NCPNN database on July 10, 2020 (IRB ID: PED.KY.01). Data from the NCPNN are only available upon request due to consent restrictions for sharing the dataset publicly.

**Fig 1:**
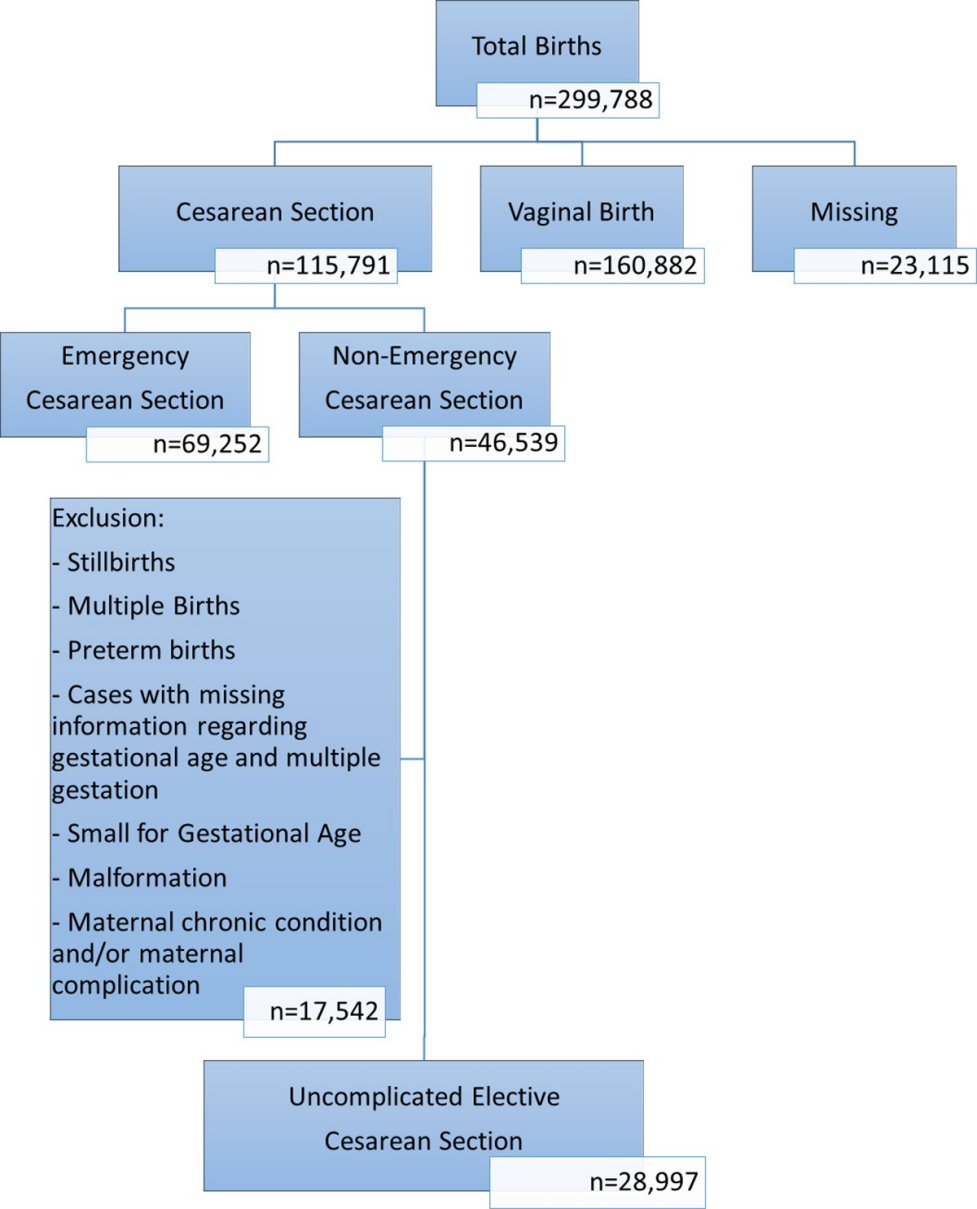
Flow chart detailing criteria applied to reach study sample.

We first determined the overall CS rate and the rate of elective CS. Our target population consisted of mothers who delivered through a non-emergency CS; i.e. term neonates delivered through uncomplicated pregnancies by low risk mothers through an elective CS including primary and repeat CS. Thus, newborns born to mothers with a chronic condition (hypertension, asthma, epilepsy, anemia, diabetes mellitus, heart disease, hypothyroidism, hyperthyroidism, and hemoglobinopathies) and/or a complication during pregnancy (urinary tract infection, bleeding, eclampsia, preeclampsia, gestational diabetes mellitus, and hospitalization during pregnancy) were excluded. Additionally, newborns born small for gestational age, from a multiple birth, and/or with a birth defect were excluded given their adverse outcomes that are unrelated to CS. Fig 1 shows the exclusions and the final sample consisting of low-risk mothers who had elective CS.

### Study variables

While the term ‘elective cesarean delivery’ is often used, it can include medical and obstetrical indications that occur before labor, or procedures for which there is no clear medical or obstetrical indication [12]. In our study, we defined the term elective CS as delivery with no labor or rupture of membrane. The documented reasons for elective CS included repeat CS, breech presentation, suspected pelvic-fetal disproportion, assisted reproductive technology, maternal age, tubal ligation, and maternal choice [12]. The study exposure was the timing of term elective CS examined separately for primary and repeat CS. Gestational age in our study was defined based on fetal ultrasound and/or last menstrual period depending on the method used by the attending obstetrician.

The neonatal adverse outcomes studied included individual and composite outcomes. The individual outcomes included respiratory distress syndrome (RDS), transient tachypnea of newborn (TTN), oxygen supplementation in the NICU, continuous positive airway pressure (CPAP), admission to the NICU, hospitalization for 5 or more days, and hyperbilirubinemia (conjugated and unconjugated). The composite outcomes were defined as the occurrence of at least one of the following reported conditions/events:

- *Resuscitation*: oxygen, bag/mask, intubation, epinephrine, cardiac compression, or sodium bicarbonate treatment
- *Pulmonary outcomes*: RDS, TTN, pneumonia (due to infection, meconium aspiration, or aspiration of other fluids), pneumothorax, bronchopulmonary dysplasia, apnea, persistent neonatal hypertension, pleural effusion, pulmonary hypoplasia, pulmonary hemorrhage, or pulmonary hypertension
- *Respiratory outcome*: pulmonary outcomes, oxygen supply in the NICU, or CPAP
- *Any outcome*: Any previously stated outcome or neonatal death (≤ 28 days after birth)

Several covariates of interest were assessed for their association between timing of elective CS and adverse neonatal outcomes. The selected covariates were based on the literature and included maternal age, parity, smoking, maternal education, and newborn sex.

### Statistical analysis

Data cleaning and analysis were conducted using SPSS statistical software version 25. Descriptive analysis was performed to assess the distribution of data using frequencies for categorical variables. Chi-square test was used to assess statistical significance. Since all the outcomes of interest were binary, simple and multivariable logistic regression models were conducted to study the association between timing of term elective CS and the adverse neonatal outcomes. In the multivariable analysis, the covariates previously mentioned were included. Statistical significance was set at the 5% level. For consistency, the unadjusted models used the same sample size as the adjusted models to account for missing data for the adjusted variables. The 39 completed weeks of gestation was used as the reference week as it’s the recommended gestational age for CS delivery with minimal adverse health outcomes [20]. In a separate analysis, we used 38 instead of 39 weeks of gestation as a reference. We also calculated the attributable risk of an adverse outcome for infants born at 37 or 38 weeks compared to infants born at 39 weeks. The attributable risk was calculated using relative risk (RR) as follows: (100 × (RR − 1) ÷ RR), where the RR is the risk at 37 or 38 weeks of gestation divided by the risk at 39 weeks of gestation.

## Results

**Fig 2:**
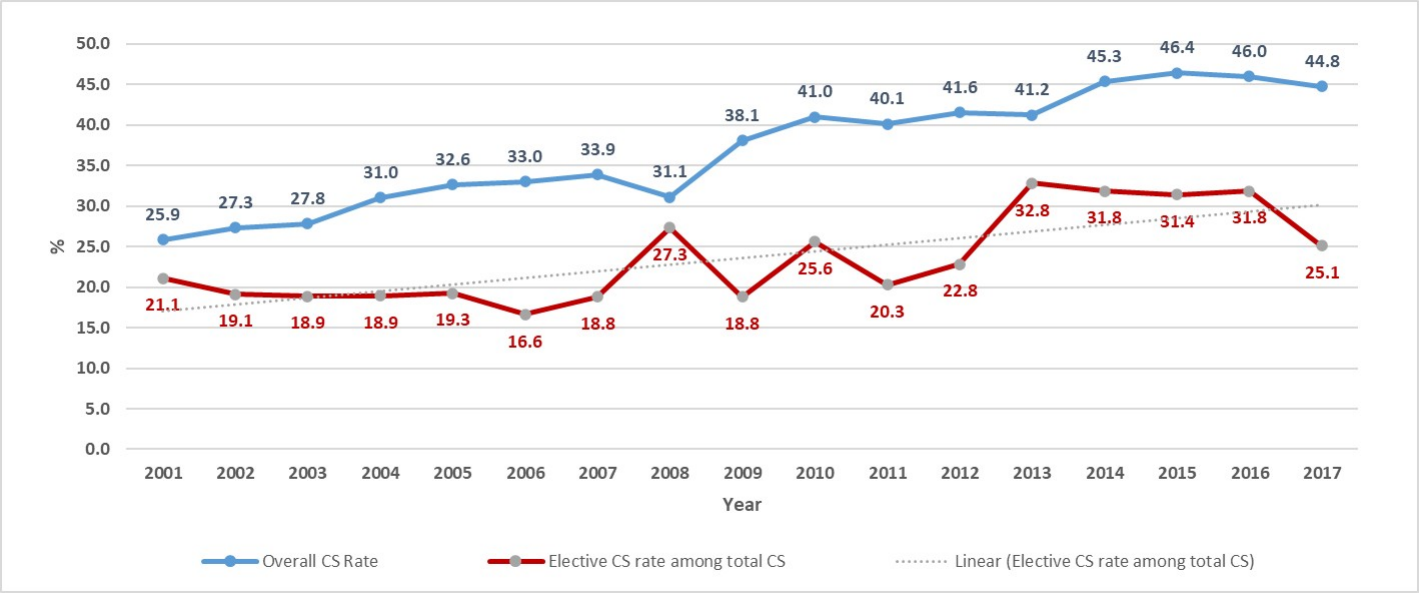
Trends of CS and elective CS between 2001 and 2017.

Between 2001 and 2017, 299,788 newborns were delivered; 115,791 by CS, out of which 69,252 were emergency and 46,539 were non-emergency. After excluding mothers with chronic conditions and/or complications during pregnancy, a total of 28,997 women (25% of all CS deliveries and 62% of uncomplicated non-emergency CS) were included in the study (Fig 1). Of the 28,997 women who had term elective cesarean section, 4,875 (16.8%) births were primary elective CS while 24,120 (83.2%) were repeat elective CS. The rate of CS in our population increased between 2001 (25.9%) and 2017 (44.8%) reaching a high of 46.4% in 2015 (Fig 2). Elective CS rate varied throughout the years from a low of 16.6% among all CS births in 2006 to a high of 32.8% in 2013.

**Fig 3:**
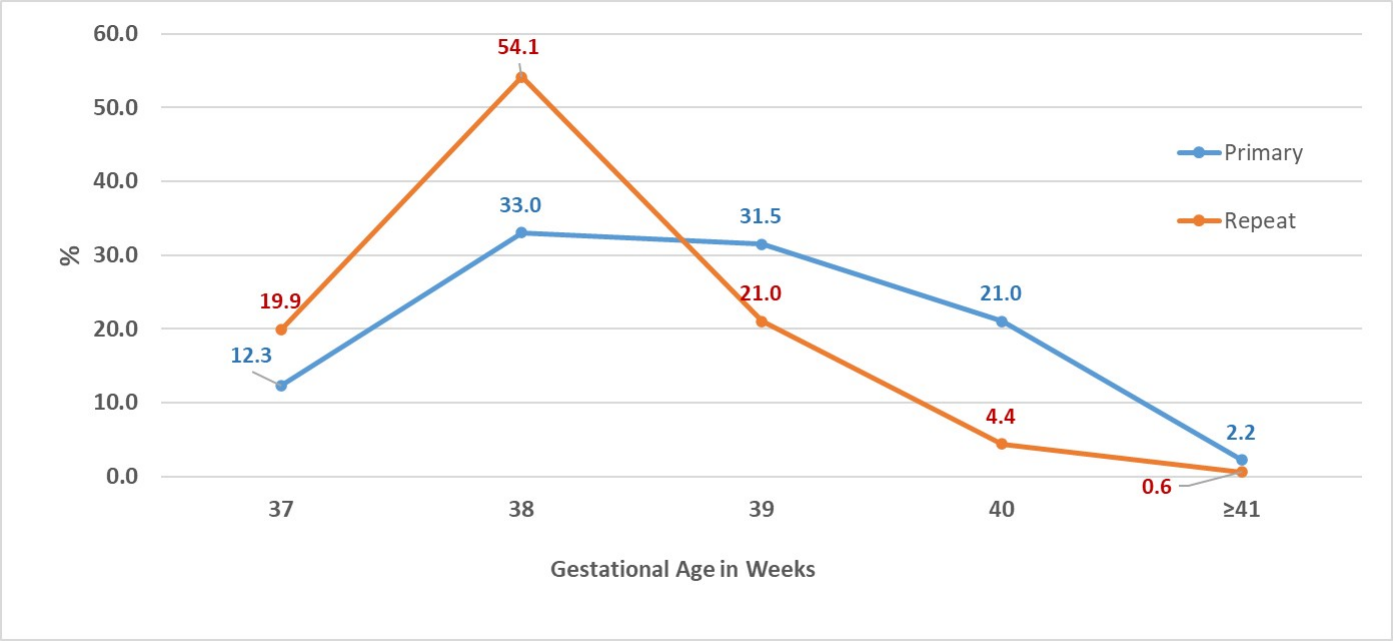
Distribution of gestational ages at delivery among primary and repeat elective CS.

**Fig 4:**
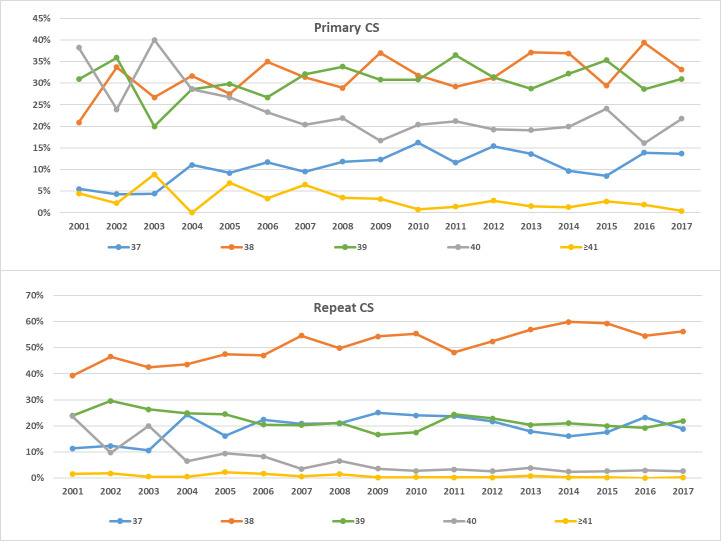
Timing of CS delivery trends from 2001 to 2017. (A) Among primary CS. (B) Among repeat CS.

The distribution of gestational ages differed between primary and repeat elective CS as shown in Fig 3 (P-value < 0.001). About a third of the women (31.5%) had their first CS at 39 weeks of gestation, while 12.3% and 33% had their CS at 37 and 38 weeks of gestation, respectively. Only 21% of women who previously had a CS, delivered at 39 weeks of gestation; 20% and 54% delivered at 37 and 38 weeks of gestation, respectively. The trends for the timing of delivery over the years had been consistent among primary and repeat CS (Fig 4A and 4B). The rates of delivery among primary and repeat CS during the 39 weeks of gestation fluctuated throughout the years around the averages of 31% and 22%, respectively. The rates of delivery during 37 and 38 weeks of gestation increased while delivery post 39 weeks decreased.

The majority of women who underwent primary and repeat CS were between 25 and 35 years of age, did not smoke, and had at least a high school education(Table 1). There were significant differences in the delivery gestational week for maternal age and parity among women who had a primary CS, and in maternal education, age, and newborn sex among women who had a repeat CS (Table 1).

**Table 1:** Maternal and infant characteristics among women with primary and repeat CS by gestational age.

	Primary CS				Repeat CS			
	37 weeks N = 598*	38 weeks N = 1,606*	39 weeks N = 1,537*	P-Value	37 weeks N = 4,796*	38 weeks N = 13,054*	39 weeks N = 5,077*	P-value
**Maternal characteristics**								
** Education**								
** Illiterate**	5 (0.9%)	30 (2.0%)	19 (1.4%)	0.055	88 (2.0%)	232 (2.0%)	95 (2.2%)	0.002
** Read/write/elementary**	77 (14.3%)	167 (11.3%)	130 (9.5%)		534 (12.1%)	1,384 (11.8%)	596 (14.1%)	
** Int/secondary/tech**	256 (47.6%)	735 (49.5%)	687 (50.3%)		2,305 (52.3%)	6,060 (51.5%)	2,086 (49.2%)	
** University**	200 (37.2%)	552 (37.2%)	529 (38.8%)		1,483 (33.6%)	4,088 (34.8%)	1,464 (34.5%)	
** Maternal Age**								
** < 25**	140 (24.7%)	393 (25.9%)	392 (27.6%)	0.001	589 (12.9%)	1,749 (14.4%)	743 (16.7%)	< 0.001
** 25–35**	295 (52.1%)	861 (56.7%)	810 (57.1%)		3,008 (65.9%)	8,168 (67.2%)	2,951 (66.3%)	
** >35**	131 (23.1%)	265 (17.4%)	216 (15.2%)		967 (21.2%)	2,230 (18.4%)	759 (17.0%)	
** Parity**								
** Primipara**	338 (57.0%)	1,013 (63.7%)	961 (63.7%)	0.010	59 (1.2%)	147 (1.1%)	75 (1.5%)	0.146
** Multipara**	255 (43.0%)	578 (36.3%)	548 (36.3%)		4,703 (98.8%)	12,796 (98.9%)	4,936 (98.5%)	
** Smoking**								
** No**	527 (88.0%)	1,415 (88.0%)	1,356 (88.2%)	0.981	4,062 (84.7%)	11,166 (85.5%)	4,318 (85.0%)	0.308
** Yes**	72 (12.0%)	193 (12.0%)	181 (11.8%)		736 (15.3%)	1,891 (14.5%)	760 (15.0%)	
**Infant characteristics**								
** Sex**								
** Male**	298 (50.7%)	776 (49.6%)	786 (52.1%)	0.380	2,413 (51.6%)	6,497 (50.9%)	2,449 (49.0%)	0.024
** Female**	290 (49.3%)	789 (50.4%)	723 (47.9%)		2,266 (48.4%)	6,273 (49.1%)	2,552 (51.0%)	

*The reported N represents the total number of women for each gestational age.

Among women who underwent primary CS, unadjusted analysis showed that birth at 37 compared to 39 weeks of gestation was associated with increased odds of resuscitation, NICU admission, RDS, CPAP, oxygen supplementation, the composite pulmonary outcome, the composite respiratory outcome, and any composite outcome (Table 2). Adjusting for newborn sex, maternal age, parity, education, and smoking maintained that delivery at 37 weeks of gestation increased the odds of resuscitation (OR = 4.5; 95% CI 1.8, 11.5), NICU admission (OR = 1.6; 95% CI 1.0, 2.5), RDS (OR = 13.6; 95% CI 1.5, 120.8), CPAP (OR = 8.3; 95% CI 1.6, 42.5), oxygen supplementation in the NICU (OR = 4.4; 95% CI 1.9, 10.0), the composite pulmonary outcome (OR = 9.5; 95% CI 2.6, 34.7), and the composite respiratory outcome (OR = 4.2; 95% CI 2.0, 9.0). Among those who were born at 38 weeks of gestation, unadjusted analysis revealed increased odds of resuscitation, oxygen supply in the NICU and the composite respiratory outcome. After adjusting for newborn sex, maternal age, parity, education, and smoking, the 2-3-fold increased odds of resuscitation, oxygen supply in the NICU, and the composite respiratory outcome remained significant (Table 2).

**Table 2:** Unadjusted and adjusted ORs for adverse neonatal outcomes at 37 and 38 compared to 39 weeks of gestation among women with primary CS.

	37 weeks			38 weeks				39 weeks
Outcomes	N/Total (%)	Unadjusted OR ^a^ (95% CI)	Adjusted OR ^b^^,^ ^c^ (95% CI)	N/Total (%)	Unadjusted OR ^a^ (95% CI)	Adjusted OR ^b^^,^ ^d^ (95% CI)	N/Total (%)	
**Post delivery**	** **	** **	** **	** **	** **	** **		
** Resuscitation**	13/516 (2.5)	4.7 (1.9, 12.0)	4.5 (1.8, 11.5)	21/1,395 (1.5)	2.8 (1.2, 6.6)	2.8 (1.2, 6.7)	7/1,292 (0.5)	
** NICU Admission**	35/516 (6.8)	1.7 (1.1, 2.5)	1.6 (1.0, 2.5)	67/1,395 (4.8)	1.2 (0.8, 1.7)	1.2 (0.8, 1.7)	54/1,292 (4.2)	
** Hospital Stay ≥ 5 days**	7/485 (1.4)	1.5 (0.6, 3.9)	1.9 (0.7, 5.2)	12/1,340 (0.9)	0.9 (0.4,2.1)	1.1 (0.5, 2.6)	12/1,249 (1)	
**Morbidities**								
** RDS**	5/516 (1.0)	12.6 (1.5, 108.2)	13.6 (1.5, 120.8)	3/1,391 (0.2)	2.8 (0.3, 26.8)	2.8 (0.3, 27.3)	1/1,290 (0.1)	
** TTN**^**e**^	0/51 (0.0)	—	—	2/182 (1.1)	—	—	0/186 (0.0)	
** Conjug. hyperb.**	9/476 (1.9)	0.9 (0.5, 2.3)	1.0 (0.5, 2.2)	20/1,217 (1.6)	0.9 (0.5,1.7)	0.9 (0.5, 1.7)	21/1,183 (1.8)	
** Unconj. hyperb.**	14/470 (3.0)	1.1 (0.6, 2.1)	1.0 (0.5, 2.0)	36/1,191 (3.0)	1.1 (0.7, 1.8)	1.1 (0.7, 1.8)	31/1,161 (2.7)	
**Management**								
** CPAP**	6/514 (1.2)	7.6 (1.5, 37.8)	8.3 (1.6, 42.5)	5/1,387 (0.4)	2.3 (0.5, 12.0)	2.5 (0.5, 12.9)	2/1,290 (0.2)	
** Oxygen**	16/514 (3.1)	4.6 (2.0, 10.4)	4.4 (1.9, 10.0)	22/1,392 (1.6)	2.3 (1.1, 5.0)	2.3 (1.0, 5.0)	9/1,290 (0.7)	
**Composite Outcomes**								
** Pulmonary**	11/516 (2.1)	9.3 (2.6, 33.6)	9.5 (2.6, 34.7)	11/1,391 (0.8)	3.4 (0.9, 12.3)	3.6 (0.9, 12.5)	3/1,290 (0.2)	
** Respiratory**	19/516 (3.7)	4.4 (2.1, 9.4)	4.2 (2.0, 9.0)	27/1,392 (1.9)	2.3 (1.1, 4.7)	2.3 (1.1, 4.7)	11/1,290 (0.9)	
** Any outcome**	42/516 (8.1)	1.5 (1.1, 2.3)	1.5 (0.9, 2.2)	89/1,395 (6.4)	1.2 (0.9, 1.6)	1.2 (0.9, 1.6)	70/1,292 (5.4)	

^a^ For consistency, the unadjusted model uses the same sample size as the adjusted model and accounts for missing data for the adjusted variables.

^b^ The reported ORs for each outcome are adjusted for newborn sex, parity, maternal age, education, and smoking.

^c^ For adjusted analysis among primary CS, a 14.4–14.8% missing rate has been reported for the outcomes resuscitation, NICU admission, hospital stay ≥ 5 days, RDS, TTN, CPAP, oxygen, pulmonary, respiratory and any outcome. While a missing rate of 22.8% and 24.3% has been reported for conjugated and unconjugated hyperbilirubinemia respectively.

^d^ For adjusted analysis among repeat CS, a 15.3–15.8% missing rate has been reported for the outcomes resuscitation, NICU admission, hospital stay ≥ 5 days, RDS, CPAP, oxygen, pulmonary, respiratory, and any outcome. While a missing rate of 13.4%, 22.8% and 24.3% has been reported for TTN, conjugated and unconjugated hyperbilirubinemia respectively.

^e^ Data on this variable are available from January 2001 to March 2008.

Among women who underwent repeat CS at 37 weeks of gestation, unadjusted analysis showed that all the examined outcomes were associated with increased odds at 37 weeks with odds ratios ranging from 1.5 to 15.7 (Table 3). Adjusting for newborn sex, maternal age, parity, education, and smoking did not change the results. Among women who underwent repeat CS, unadjusted and adjusted analyses showed that birth at 38 weeks of gestation was associated with RDS (Table 3).

**Table 3:** Unadjusted and adjusted ORs for adverse neonatal outcomes at 37 and 38 compared to 39 weeks of gestation among women with repeat CS.

	37 weeks			38 weeks				39 weeks
Outcomes	N (%)	Unadjusted OR (95% CI) ^a^	Adjusted OR (95% CI) ^b^^,^ ^c^	N (%)	Unadjusted OR (95% CI) ^a^	Adjusted OR (95% CI) ^b^^,^ ^d^	N (%)	
**Post delivery**	** **	** **	** **	** **	** **	** **		
** Resuscitation**	133/4,198 (3.2)	2.4 (1.8, 3.3)	2.5 (1.8, 3.4)	195/11,226 (1.7)	1.3 (0.9, 1.9)	1.3 (0.9, 1.8)	54/4,069 (1.3)	
** NICU admission**	341/4,198 (8.1)	1.8 (1.5, 2.1)	1.8 (1.5, 2.1)	517/11,226 (4.6)	0.9 (0.8, 1.1)	1.0 (0.8, 1.1)	195/4,069 (4.8)	
** Hospital stay ≥ 5 days**	91/3,936 (2.3)	1.5 (1.1, 2.1)	1.8 (1.3, 2.6)	110/10,826 (1.0)	0.7 (0.5, 0.9)	0.9 (0.6, 1.2)	60/3,936 (1.5)	
**Morbidities**								
** RDS**	32/4,169 (0.8)	15.7 (3.8, 65.5)	15.8 (3.8, 65.9)	31/11,187 (0.3)	5.6 (1.3, 23.6)	5.6 (1.4, 23.5)	2/4,060 (0)	
** TTN**^**e**^	13/637 (2.0)	3.4 (1.2, 9.5)	3.6 (1.3, 10.1)	19/1650 (1.2)	1.9 (0.7, 5.1)	2.0 (0.7, 5.3)	5/815 (0.6)	
** Conjug. hyperb.**	118/3,797 (3.1)	1.6 (1.2, 2.1)	1.6 (1.2, 2.1)	207/10,117 (2.0)	1.0 (0.8, 1.3)	1.1 (0.8, 2.1)	75/3,768 (2)	
** Unconj. hyperb.**	187/3,685 (5.1)	1.5 (1.2, 1.9)	1.5 (1.2, 1.9)	322/9,934 (3.2)	0.9 (0.8, 1.1)	0.9 (0.8, 1.2)	128/3,672 (3.5)	
**Management**								
** CPAP**	45/4,162 (1.1)	2.8 (1.6, 4.9)	2.8 (1.6, 5.0)	46/11,187 (0.4)	1.0 (0.6, 1.8)	1.1 (0.6, 1.9)	16/4,054 (0.4)	
** Oxygen**	159/4,172 (3.8)	2.5 (1.9, 3.4)	2.6 (1.9, 3.5)	216/11,197 (1.9)	1.3 (0.9, 1.7)	1.3 (0.9, 1.7)	63/4,059 (1.6)	
**Composite Outcomes**								
** Pulmonary**	87/4,172 (2.1)	2.8 (1.8, 4.2)	2.7 (1.8, 4.1)	96/11,187 (0.9)	1.1 (0.7, 1.7)	1.1 (0.7, 1.7)	31/4,060 (0.8)	
** Respiratory**	182/4,175 (2.4)	2.6 (2.0, 3.4)	2.6 (2.0, 3.5)	246/11,204 (2.2)	1.3 (0.9, 1.8)	1.3 (0.9, 1.7)	70/4,060 (1.7)	
** Any outcome**	456/4,198 (10.9)	1.6 (1.4, 1.9)	1.6 (1.4, 1.9)	714/11,226 (6.4)	0.9 (0.8, 1.1)	0.9 (0.8, 1.1)	281/4,069 (6.9)	

^a^ For consistency, the unadjusted model uses the same sample size as the adjusted model and accounts for missing data for the adjusted variables.

^b^ The reported ORs for each outcome are adjusted for newborn sex, parity, maternal age, education, and smoking.

^c^ For adjusted analysis among primary CS, a 14.4–14.8% missing rate has been reported for the outcomes resuscitation, NICU admission, Hospital Stay ≥ 5 days, RDS, TTN, CPAP, oxygen, pulmonary, respiratory and any outcome. While a missing rate of 22.8% and 24.3% has been reported for conjugated and unconjugated hyperbilirubinemia respectively.

^d^ For adjusted analysis among repeat CS, a 15.3–15.8% missing rate has been reported for the outcomes resuscitation, NICU admission, Hospital Stay ≥ 5 days, RDS, CPAP, oxygen, pulmonary, respiratory, and any outcome. While a missing rate of 13.4%, 22.8% and 24.3% has been reported for TTN, conjugated and unconjugated hyperbilirubinemia respectively.

^e^ Data on this variable are available from January 2001 to March 2008.

Although 39 weeks of gestation is considered the reference week, we also assessed the odds of adverse outcomes of primary and repeat CS at 37 weeks of gestation compared to 38 weeks, since delivery at 38 weeks represents the most common week of delivery at 50.5%. Adjusted analysis showed that among women who had primary CS, delivery at 37 compared to 38 weeks of gestation significantly increased the odds for RDS (OR = 4.8; 95% CI 1.1, 21.1) and the composite pulmonary outcome (OR = 2.7; 95% CI 1.2, 6.5) (Table 4). Delivery at 37 compared to 38 weeks of gestation among repeat CS significantly increased the odds of all adverse outcomes, except for TTN (Table 4).

**Table 4:** Unadjusted and adjusted ORs for adverse neonatal outcomes at 37 compared to 38 weeks of gestation among women with primary or repeat CS.

	Primary			Repeat		
Outcomes	N/Total (%)	Unadjusted OR ^a^ (95% CI)	Adjusted OR ^b^^,^ ^c^ (95% CI)	N (%)	Unadjusted OR ^a^ (95% CI)	Adjusted OR ^b^^,^ ^d^ (95% CI)
**Post delivery**	** **	** **	** **	** **	** **	** **
** Resuscitation**	13/516 (2.5)	1.7 (0.8, 3.4)	1.6 (0.8, 3.3)	133/4,198 (3.2)	1.9 (1.5, 2.3)	1.9 (1.5, 2.3)
** NICU Admission**	35/516 (6.8)	1.4 (0.9, 2.2)	1.4 (0.9, 2.1)	341/4,198 (8.1)	1.8 (1.6, 2.1)	1.8 (1.6, 2.1)
** Hospital Stay ≥ 5 days**	7/485 (1.4)	1.6 (0.6, 4.1)	1.6 (0.6, 4.1)	91/3,936 (2.3)	2.3 (1.7, 3.1)	2.3 (1.7, 3.0)
**Morbidities**						
** RDS**	5/516 (1.0)	4.5 (1.1, 19.0)	4.8 (1.1, 21.1)	32/4,169 (0.8)	2.8 (1.7, 4.6)	2.8 (1.7, 4.6)
** TTN**^**e**^	0/51 (0.0)	—	—	13/637 (2.0)	1.8 (0.9, 3.6)	1.8 (0.9, 3.7)
** Conjug. hyperb.**	9/476 (1.9)	1.2 (0.5, 2.6)	1.1 (0.5, 2.4)	118/3,797 (3.1)	1.5 (1.2, 1.9)	1.5 (1.2, 1.9)
** Unconj. hyperb.**	14/470 (3.0)	0.9 (0.5, 1.8)	0.9 (0.5, 1.8)	187/3,685 (5.1)	1.6 (1.3, 1.9)	1.6 (1.3, 1.9)
**Management**						
** CPAP**	6/514 (1.2)	3.3 (0.9, 10.7)	3.4 (0.9, 11.3)	45/4,162 (1.1)	2.6 (1.8, 4.0)	2.7 (1.8, 4.0)
** Oxygen**	16/514 (3.1)	2.0 (1.1, 3.8)	1.9 (0.9, 3.7)	159/4,172 (3.8)	2.0 (1.6, 2.5)	2.0 (1.7, 2.5)
**Composite Outcomes**						
** Pulmonary**	11/516 (2.1)	2.7 (1.2, 6.3)	2.7 (1.2, 6.5)	87/4,172 (2.1)	2.5 (1.8, 3.3)	2.5 (1.8, 3.3)
** Respiratory**	19/516 (3.7)	1.9 (1.1, 3.5)	1.8 (0.9, 3.4)	182/4,175 (2.4)	2.0 (1.7, 2.5)	2.0 (1.7, 2.5)
** Any outcome**	42/516 (8.1)	1.3 (0.9, 1.9)	1.3 (0.9, 1.8)	456/4,198 (10.9)	1.8 (1.6, 2.0)	1.8 (1.6, 2.0)

^a^ For consistency, the unadjusted model uses the same sample size as the adjusted model and accounts for missing data for the adjusted variables.

^b^ The reported ORs for each outcome are adjusted for newborn sex, parity, maternal age, education, and smoking.

^c^ For adjusted analysis among primary CS, a 14.4–14.8% missing rate has been reported for the outcomes resuscitation, NICU admission, Hospital Stay ≥ 5 days, RDS, TTN, CPAP, oxygen, pulmonary, respiratory and any outcome. While a missing rate of 22.8% and 24.3% were reported for conjugated and unconjugated hyperbilirubinemia respectively.

^d^ For adjusted analysis among repeat CS, a 15.3–15.8% missing rate has been reported for the outcomes resuscitation, NICU admission, Hospital Stay ≥ 5 days, RDS, CPAP, oxygen, pulmonary, respiratory and any outcome. While a missing rate of 13.4%, 22.8% and 24.3% were reported for TTN, conjugated and unconjugated hyperbilirubinemia respectively.

^e^ Data on this variable is available from January 2001 to March 2008.

Calculating the attributable risk of having an adverse outcome due to repeat elective delivery at 37 weeks of gestation reveals that postponing delivery to 39 weeks might prevent 42% of cases of any adverse outcome, 64% of cases of the combined adverse respiratory outcome, 94% of RDS cases, 62% of cases of resuscitation, and 46% of cases of NICU admission. Concurrently, postponing elective repeat delivery from 38 to 39 weeks of gestation might prevent 26% of cases of the composite adverse respiratory outcome, and 83% of RDS cases. For primary elective CS, postponing delivery from 37 to 39 weeks might prevent 77% of adverse respiratory outcome cases, 92% of RDS cases, 79% resuscitation, and 40% of NICU admissions. While postponing delivery from 38 to 39 weeks, might prevent 57% of adverse respiratory outcome cases.

## Discussion

In this multi-center study, uncomplicated elective planned CS constituted 25% of all CS deliveries and 9.7% of all deliveries between 2001 and 2017. Our rate of elective CS lies within the average reported among European countries and other Middle Eastern countries [6–9,21]. Of those non-emergency CS, 62% were uncomplicated CS. More than two thirds of these deliveries were performed before 39 weeks of gestation.

To our knowledge, this is the first study to assess timing of elective CS and adverse neonatal outcomes in the region [8,9,21]. Moreover, this study assesses both primary and repeat elective CS instead of studying the overall elective CS population [11,14,15,17] or repeat CS only [10,12,13,21,22]. Our results are also in line with those of other studies that showed that delivery at 37 compared to 39 weeks of gestation is associated with an increased odds of adverse respiratory outcomes, admission to the NICU, and hospital stay for more than 5 days. A prospective study conducted at 19 US academic centers showed that among early term elective repeat CS, NICU admission, TTN, as well as respiratory distress syndrome were the most common adverse outcomes seen at all gestational ages, but more commonly at 37 weeks of gestation compared to 39 weeks of gestation [22]. Another retrospective cohort study in China revealed that delivery at 37 compared to 39 weeks of gestation was associated with increased odds of neonatal respiratory disease, hyperbilirubinemia, NICU admission, and prolonged hospitalization [17].

The high rate of CS in Lebanon is mainly due to a privatized health care system dominated by the private health insurance system, the lack of physician accountability, the diminishing role of midwives, as well as women’s misconceptions on both vaginal and CS procedures and safety [23,24]. In the presence of several factors affecting the decision to carry out a CS, national and hospital policies are needed for safer CS delivery practices. Unfortunately, there are no existing policies or legislative regulations to decrease the rate of CS in Lebanon [25]. One of the main barriers is the dominance of the private health sector over the public authorities such as the Ministry of Public Health to effectively establish new standards of health services. Despite the urgent need to reduce rates of unnecessary CS in Lebanon, the Order of Physicians and most of the obstetricians in Lebanon are against standardizing guidelines to reduce the rate of CS [25]. One of the recommendations to decrease the rate of CS is to enforce physicians to obtain a second medical opinion before proceeding with a CS [25]. Another recommendation is to implement monthly reviews of labor and delivery records to assess the indications for the performed CS and the quality of maternal care [25]. Aside from the urgent need to reduce the rate of elective CS in Lebanon given the associated neonatal and maternal complications [26,27], other simple strategies might just include delaying birth by 1–2 weeks till 39 weeks of gestation to at least decrease the rate of neonatal morbidities.

In the U.S., several studies have shown that it is possible to reduce non-medically indicated early term deliveries. Clark et al. (2010) have shown that a strict “hard stop” hospital policy is the most effective intervention for decreasing elective CS without any effect on stillbirth rates when compared to physician education or adoption of a “soft stop”policy requiring a local evaluation and decision making by a peer review committee [28]. Clark et al. demonstrated that creating and enforcing hospital policies that ban elective deliveries prior to 39 weeks of gestation decreased early term deliveries from 8.2% to 1.7% (P-value = 0.007) [28]. Moreover, state-wide hard-stop policies implemented in South Carolina and Oregon to limit early elective deliveries have shown a respective 50% and 38% decrease in early elective CS [29,30].

### Limitations of the data

While our results found odds ratios in the same direction as that reported in the literature at 38 weeks of gestation among primary and repeat CS, our measures of association did not reach statistical significance [12,14]. The different patterns of association in our data might be due to restricting our analysis to uncomplicated elective CS due to our inability to determine whether CS among women with pregnancy complications was elective. Thus, we might have missed some elective cases. However, limiting our analysis to uncomplicated pregnancies ensures that our studied sample reflects the association between timing of elective CS and adverse neonatal outcomes without risking the confounding effects of chronic and pregnancy complications among mothers. Other possible limitations include the non-uniformity in identifying gestational age between physicians (last menstrual period vs. ultrasound). Another limitation is that the NCPNN is not a national registry but covered between 2001 and 2017 around 21% of all deliveries in Lebanon potentially affecting the generalizability of our findings.

## Conclusions

Since Lebanon has a high number of CS, the determinants leading to elective CS prior to 39 weeks of gestation are in dire need of assessment. Further qualitative and quantitative studies are needed to better understand the factors leading to an elective CS in Lebanon to set the stage for appropriate interventions to decrease the CS rate.

## Supporting information

S1 Checklist STROBE statement—checklist of items that should be included in reports of observational studies. (DOC)
